# Supplementary material for: Controlling Microparticle Morphology in Melt-Jet Printing of Active Pharmaceutical Ingredients through Surface Phenomena
Source: Pharmaceutics. 2023 Jul 26;15(8):2026. doi: 10.3390/pharmaceutics15082026 (PMC10459835; doi:10.3390/pharmaceutics15082026)
Supplement: Supplementary file 1 [file pharmaceutics-15-02026-s001.zip › pharmaceutics-2473875-supplementary.pdf]

Supplementary Materials

Table S1: Surface roughness (Ra) measurements by AFM.

**Surface**	**Ra**
PTFE	24.6 ± 8.1
Aluminum	118.9 ± 23.6
PE	218.1 ± 39.8
Glass	13.6 ± 1.8

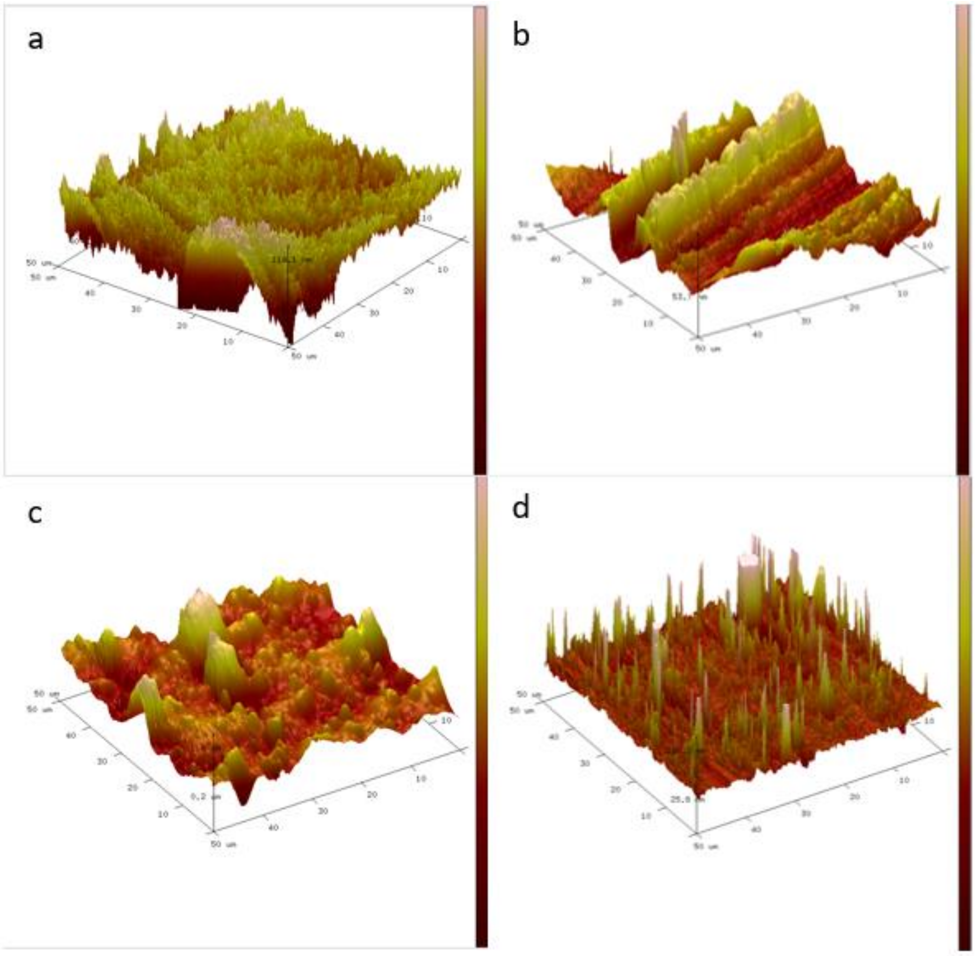

Figure S1. Three-dimensional AFM images of (a) PTFE, (b) aluminum, (c) PE, and (d) glass surfaces.

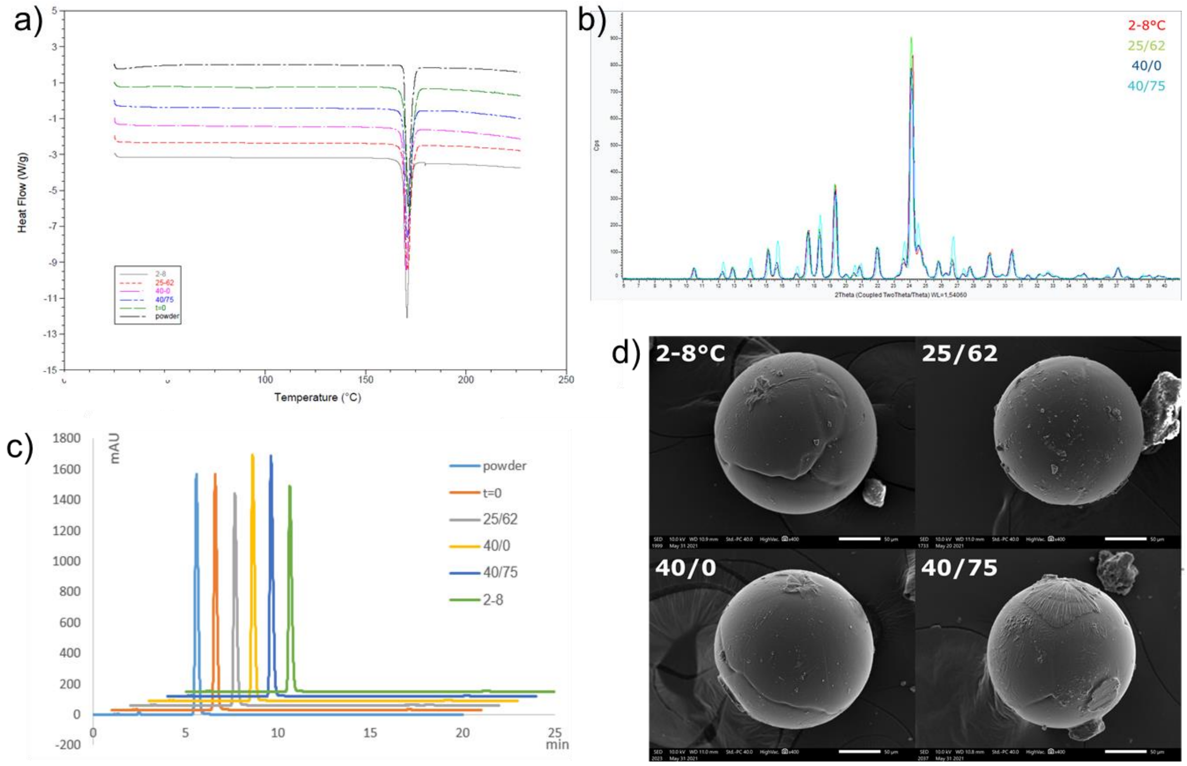

Figure S2. Paracetamol particle stability analysis after 4 weeks of storage. a) DSC thermograms, b) XRD patterns, c) HPLC chromatograms, and d) SEM images (Scale bars: 50µm) of neat powder and printed particles stored under different conditions: 2-8°C, 25°C/62% RH, 40°C /0% RH, and 40°C /75% RH.
